# Protective Effect of Maternal First-Trimester Low Body Mass Index Against Macrosomia: A 10-Year Cross-Sectional Study

**DOI:** 10.3389/fendo.2022.805636

**Published:** 2022-02-10

**Authors:** Yongqing Sun, Man Zhang, Ruixia Liu, Jingjing Wang, Kai Yang, Qingqing Wu, Wentao Yue, Chenghong Yin

**Affiliations:** ^1^ Prenatal Diagnosis Center, Beijing Obstetrics and Gynecology Hospital, Capital Medical University, Beijing, China; ^2^ Beijing Maternal and Child Health Care Hospital, Beijing, China; ^3^ Central Laboratory, Beijing Obstetrics and Gynecology Hospital, Capital Medical University, Beijing, China; ^4^ Department of Ultrasound, Beijing Obstetrics and Gynecology Hospital, Capital Medical University, Beijing, China

**Keywords:** macrosomia, body mass index, gestational diabetes mellitus, parity, maternal age, fetal sex, season of delivery

## Abstract

**Objective:**

We aimed to assess whether maternal first-trimester low body mass index (BMI) has a protective effect against macrosomia.

**Methods:**

This was a cross-sectional study from January 1, 2011, to June 30, 2021, and 84,900 participants were included. The predictive performance of maternal first-trimester and parental pre-pregnancy BMI for macrosomia was assessed using the area under the receiver-operating characteristics curve (AUC). Multivariate logistic regression analyses were performed to evaluate the independent effect of maternal first-trimester low BMI on macrosomia. Interactions were investigated to evaluate the potential variation of the effect of first-trimester low BMI across different groups. Furthermore, interactions were also examined across groups determined by multiple factors jointly: a) gestational diabetes mellitus (GDM)/GDM history status, parity, and maternal age; and b) GDM/GDM history status, fetal sex, and season of delivery.

**Results:**

The proportion of macrosomia was 6.14% (5,215 of 84,900). Maternal first-trimester BMI showed the best discrimination of macrosomia (all Delong tests: *P* < 0.001). The protective effect of maternal first-trimester low BMI against macrosomia remained significant after adjusting for all confounders of this study [adjusted odds ratios (aOR) = 0.37, 95% CI: 0.32–0.43]. Maternal first-trimester low BMI was inversely associated with macrosomia, irrespective of parity, fetal sex, season of delivery, maternal age, and GDM/GDM history status. The protective effect was most pronounced among pregnant women without GDM/GDM history aged 25 to 29 years old, irrespective of parity (multipara: aOR = 0.32, 95% CI: 0.22–0.47; nullipara: aOR = 0.32, 95% CI: 0.24–0.43). In multipara with GDM/GDM history, the protective effect of low BMI was only observed in the 30- to 34-year-old group (aOR = 0.12, 95% CI: 0.02–0.86). For pregnant women without GDM/GDM history, the protective effect of maternal first-trimester low BMI against macrosomia was the weakest in infants born in winter, irrespective of fetal sex (female: aOR = 0.45, 95% CI: 0.29–0.69; male: aOR = 0.39, 95% CI: 0.28–0.55).

**Conclusion:**

Maternal first-trimester low BMI was inversely associated with macrosomia, and the protective effect was most pronounced among 25- to 29-year-old pregnant women without GDM/GDM history and was only found among 30- to 34-year-old multipara with GDM/GDM history. The protective effect of maternal first-trimester low BMI against macrosomia was the weakest in winter among mothers without GDM/GDM history.

## Introduction

Fetal macrosomia is defined as a birth weight of more than 4,000 g regardless of gestational age (1). Macrosomia is an important risk factor for fetal fracture, perinatal asphyxia, cerebral hemorrhage, shoulder dystocia, and even death and is associated with long-term adverse outcomes such as obesity and cardiovascular and metabolic complications in the long run (1–3). Besides, macrosomia can also cause a number of adverse maternal health issues, such as cesarean delivery, perineal lacerations, postpartum hemorrhage, and diabetes or gestational diabetes mellitus (GDM) (1, 4). The incidence of macrosomia has been reported between 7.8% (5) and 15.1% (6) in developed countries and 7.0% in China (6). Given the immense burden of macrosomia on women and their offspring, thoroughly exploring the factors of macrosomia is of paramount importance for the development of primary preventive strategies.

There has been evidence on the association between pre-pregnancy overweight/obesity and macrosomia (7, 8). A previous study of 19,622 singleton pregnancies suggested that maternal GDM status increased the risk of macrosomia independently and synergistically with pre-pregnancy overweight (9). Despite widespread screening and management of GDM, the prevalence of macrosomia remains relatively high among pregnant women without GDM, which indicates that some other factors may also influence the incidence of macrosomia (10). It is reported that advanced maternal age (11), male fetuses (12), higher parity (13), and certain season of delivery (14) were also associated with increased odds for fetal macrosomia.

Whether pre-pregnancy low body mass index (BMI) also influence the risk of macrosomia is inconclusive until recently (15, 16). No study has reported on the effect of maternal first-trimester low BMI on macrosomia stratified by all the above risk factors. We speculated that the impact of maternal first-trimester low BMI on macrosomia may differ by these maternal factors. The aim, therefore, of this study was to determine the relationship between maternal first-trimester low BMI and macrosomia, stratifying by parity, fetal sex, season of delivery, maternal age, and GDM/GDM history status.

## Materials and Methods

### Study Design and Data Sources

**Figure 1:**
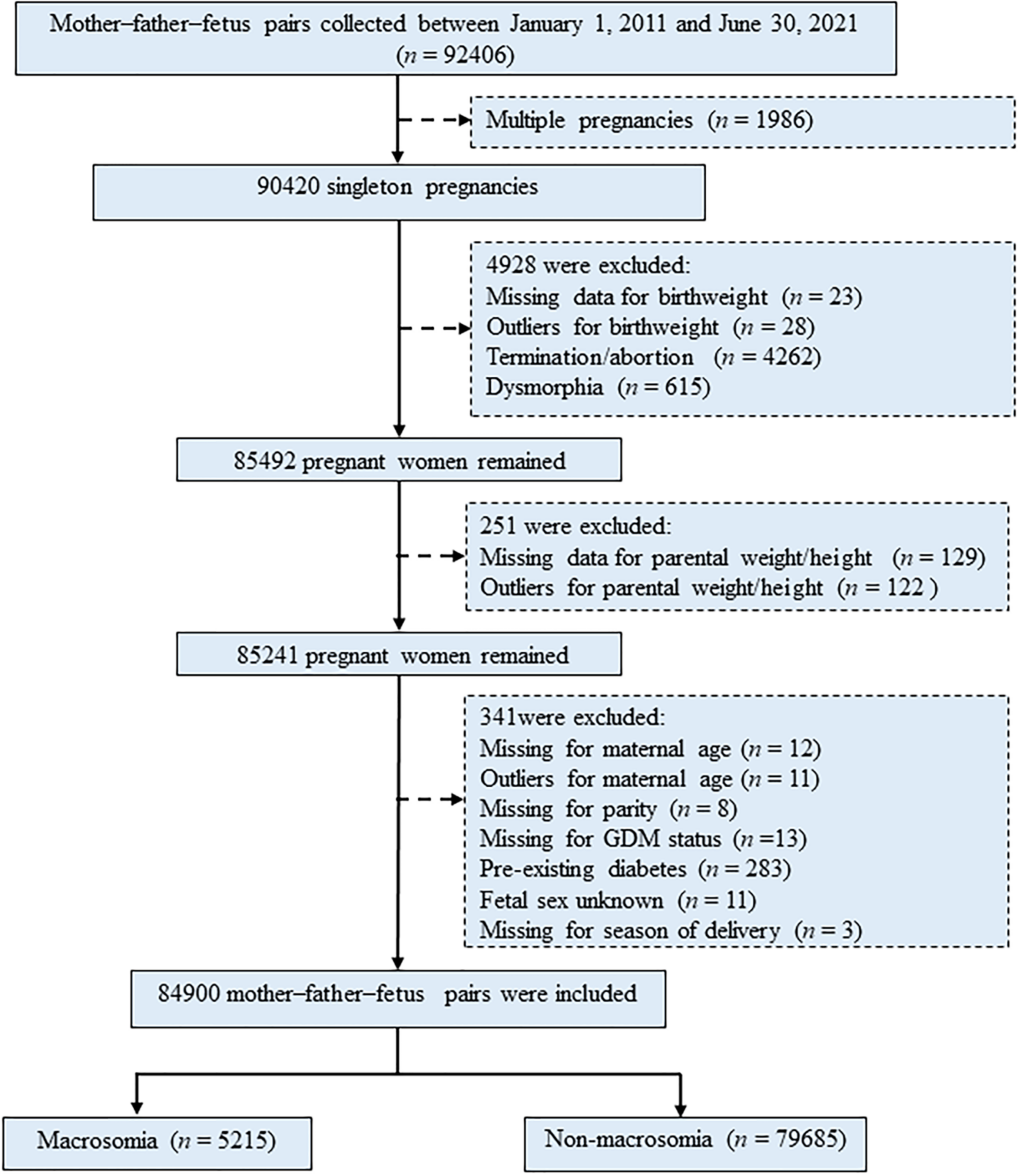
Flowchart of the selection of study participants. GDM, gestational diabetes mellitus.

Pregnant women (*n* = 92,406) were initially recruited to this study during their first prenatal visit in Beijing Obstetrics & Gynecology Hospital, Maternal & Child Health Centre, Capital Medical University, Beijing, China, from January 1, 2011, to June 30, 2021. Retrospective data of participants were retrieved from the hospital information systems, which provide comprehensive information covering results of pre-pregnancy, regular health examinations during pregnancy, and delivery details. Participants (mother–father–fetus pairs) and their detailed information at each gestation stage were identified and extracted using their unique identification (ID) numbers. Exclusion criteria included multiple pregnancies (*n* = 1,986), participants with missing data or outliers for birth weight (*n* = 51), termination/abortion (*n* = 4,262), and dysmorphia (*n* = 615), with missing data or outliers for parental weight/height (*n* = 251), with missing data or outliers for key variables (including maternal age, parity, GDM/GDM history status, fetal sex, and season of delivery) and pre-existing diabetes (*n* = 341). Thus, 84,900 singleton pregnancies were eligible for inclusion in the study for final analysis (**Figure 1**). Participation in the study was voluntary, and written informed consent was obtained from each study subject. This study was approved by the Ethical Committee of Beijing Obstetrics and Gynecology Hospital, Capital Medical University (Reference No. IEC-C-03-V04-FJ2).

### Data Collection and Measurements

For each of the 84,900 participants, four main categories of detailed information were collected: a) demographic characteristics, including maternal age, parental height, parental weight, parental ethnicity, parental education level, parental occupational physical activity, and maternal income; b) lifestyle behaviors, parental drinking, smoking, and maternal secondhand smoke exposure; c) GDM/GDM history, hypertension/hypertension history, and thyroid disease/thyroid disease history; and d) current pregnancy and other information, including conception method, parity, folic acid and multivitamin supplements, preterm birth, fetal sex, birth weight, season of delivery.

The primary outcome was macrosomia, which was defined as an infant with birth weight ≥4,000 g. Parental pre-pregnancy BMI was calculated based on self-reported values of height and weight before conception. As for pregnant women in the first trimester, the value of weight was accurately measured with electronic scales (BW-150; UWE, Beijing, China) with mothers wearing light clothes, no shoes, and empty pockets (17). Standing height was measured to the nearest 0.1 cm using a stadiometer (18). BMI was calculated as the weight in kilograms divided by the square of the height in meters. BMI was categorized as low (BMI < 18.5 kg/m^2^), normal (BMI 18.5–24.9 kg/m^2^), overweight (BMI 25.0–29.9 kg/m^2^), and obesity (BMI ≥ 30.0 kg/m^2^) based on the World Health Organization (WHO) criteria. Occupational physical activities were categorized into three levels: a) light (e.g., unemployed/housewife), b) moderate (e.g., salespeople or clerk), and c) active (e.g., farmer or manual worker) (19). Parental smoking or drinking was defined as mothers or fathers who smoked at least one cigarette per day or drank alcohol once a week for over 6 months (20). Maternal secondhand smoke exposure was defined as non-smokers being exposed to tobacco smoke of another person for at least 15 min daily for more than 1 day per week (21). GDM was diagnosed in accordance with the International Association of Diabetes and Pregnancy Study Group recommendation (IADPSG) using 75 g 2-h OGTT: a fasting glucose ≥5.1 mmol/L, or a 1-h result ≥10.0 mmol/L, or a 2-h result ≥8.5 mmol/L (22).

### Statistical Analysis

Differences for numerical and for categorical variables were calculated based on the *t*-test, Mann–Whitney *U* test, or chi-square test, as appropriate. The predictive performance of maternal first-trimester and parental pre-pregnancy BMI for macrosomia was assessed using the area under the receiver-operating characteristics curve (AUC). The DeLong test was used to compare AUCs. Univariate logistic regression analyses were used to evaluate factors that influenced macrosomia. Multivariate logistic regression analyses were performed and adjusted odds ratios (aOR) with 95% confidence intervals (CI) were calculated to evaluate the independent effect of maternal first-trimester low BMI on macrosomia. According to the recommendation of the STROBE statement, unadjusted, minimally adjusted, and fully adjusted analyses were performed. Interactions between the first-trimester low BMI and multiple individual characteristic factors were investigated to evaluate the potential variation of the effect of first-trimester low BMI across different groups. Furthermore, interactions were also examined across groups determined by multiple factors jointly: a) GDM/GDM history status, parity, and maternal age; and b) GDM/GDM history status, fetal sex, and season of delivery. *P*-values of two-sided tests less than 0.05 were considered statistically significant. All analyses were performed by R version 4.1.1 (http://www.R-project.org).

## Results

### Characteristics of Participants

Characteristics of participants are presented in **Table 1** and **Table S1**. A total of 84,900 singleton pregnancies including 5,215 (6.14%) macrosomia were ultimately included in this study. Compared with maternal first-trimester normal, overweight, and obesity BMI groups, pregnant women in the low BMI group were younger (mean age of 28.53 years old), tended to be nullipara (58.26%), less likely to deliver a macrosomia (2.12%), and had lower probability of GDM/GDM history (5.05%), hypertension/hypertension history (5.17%), and thyroid disease/thyroid disease history (8.09%).

**Table 1:** Characteristics of the participants.

Characteristics	Maternal first-trimester body mass index				*P*
	Normal	Low	Overweight	Obesity	
Maternal age (years)	30.25 (4.16)	28.53 (3.85)	30.99 (4.48)	30.55 (4.68)	<0.001
Maternal ethnicity, *n* (%)					0.482
Han	58,209 (93.86)	8,267 (93.66)	11,159 (93.65)	1,992 (93.17)	
Minority	3,810 (6.14)	560 (6.34)	757 (6.35)	146 (6.83)	
Macrosomia, *n* (%)					<0.001
No	58,514 (94.35)	8,640 (97.88)	10,661 (89.47)	1,870 (87.46)	
Yes	3,505 (5.65)	187 (2.12)	1,255 (10.53)	268 (12.54)	
Parity, *n* (%)					<0.001
Multipara	31,648 (51.03)	3,684 (41.74)	7,046 (59.13)	1,288 (60.24)	
Nullipara	30,371 (48.97)	5,143 (58.26)	4,870 (40.87)	850 (39.76)	
GDM/GDM history, *n* (%)					<0.001
No	56,262 (90.72)	8,381 (94.95)	9,955 (83.54)	1,695 (79.28)	
Yes	5,757 (9.28)	446 (5.05)	1,961 (16.46)	443 (20.72)	
Season of delivery, *n* (%)					<0.001
Spring	11,637 (18.76)	1,657 (18.77)	2,282 (19.15)	433 (20.25)	
Summer	16,907 (27.26)	2,102 (23.82)	3,400 (28.53)	611 (28.58)	
Autumn	17,385 (28.03)	2,515 (28.49)	3,303 (27.72)	571 (26.71)	
Winter	16,090 (25.95)	2,553 (28.92)	2,931 (24.60)	523 (24.46)	
Fetal sex, *n* (%)					0.007
Male	32,408 (52.25)	4,452 (50.44)	6,271 (52.63)	1,098 (51.36)	
Female	29,611 (47.75)	4,375 (49.56)	5,645 (47.37)	1,040 (48.64)	
Mode of conception, *n* (%)					<0.001
Natural conception	59,712 (96.28)	8,589 (97.30)	11,310 (94.91)	2,028 (94.86)	
Assisted reproduction	2,307 (3.72)	238 (2.70)	606 (5.09)	110 (5.14)	
Folic acid supplementation, *n* (%)					<0.001
No	4,507 (7.27)	663 (7.51)	855 (7.18)	184 (8.61)	
Yes	57,512 (92.73)	8,164 (92.49)	11,061 (92.82)	1,954 (91.39)	
Multivitamin supplementation, *n* (%)					<0.001
No	22,212 (35.81)	3,473 (39.35)	4,267 (35.81)	859 (40.18)	
Yes	39,807 (64.19)	5,354 (60.65)	7,649 (64.19)	1,279 (59.82)	
Gestational hypertension/gestational hypertension history, *n* (%)					<0.001
No	56,993 (91.90)	8,371 (94.83)	10,204 (85.63)	1,668 (78.02)	
Yes	5,026 (8.10)	456 (5.17)	1,712 (14.37)	470 (21.98)	
Thyroid disease/thyroid disease history, *n* (%)					<0.001
No	56,397 (90.94)	8,113 (91.91)	10,730 (90.05)	1,924 (89.99)	
Yes	5,622 (9.06)	714 (8.09)	1,186 (9.95)	214 (10.01)	

Data are given as mean (SD), or n (%). Normal, BMI of 18.5–24.9 kg/m^2^. Low, BMI <18.5 kg/m^2^. Overweight, BMI of 25.0–29.9 kg/m^2^. Obesity, BMI ≥30.0 kg/m^2^.

GDM, gestational diabetes mellitus.

### Comparison of Prediction Performance of Three BMIs for Macrosomia

**Figure 2:**
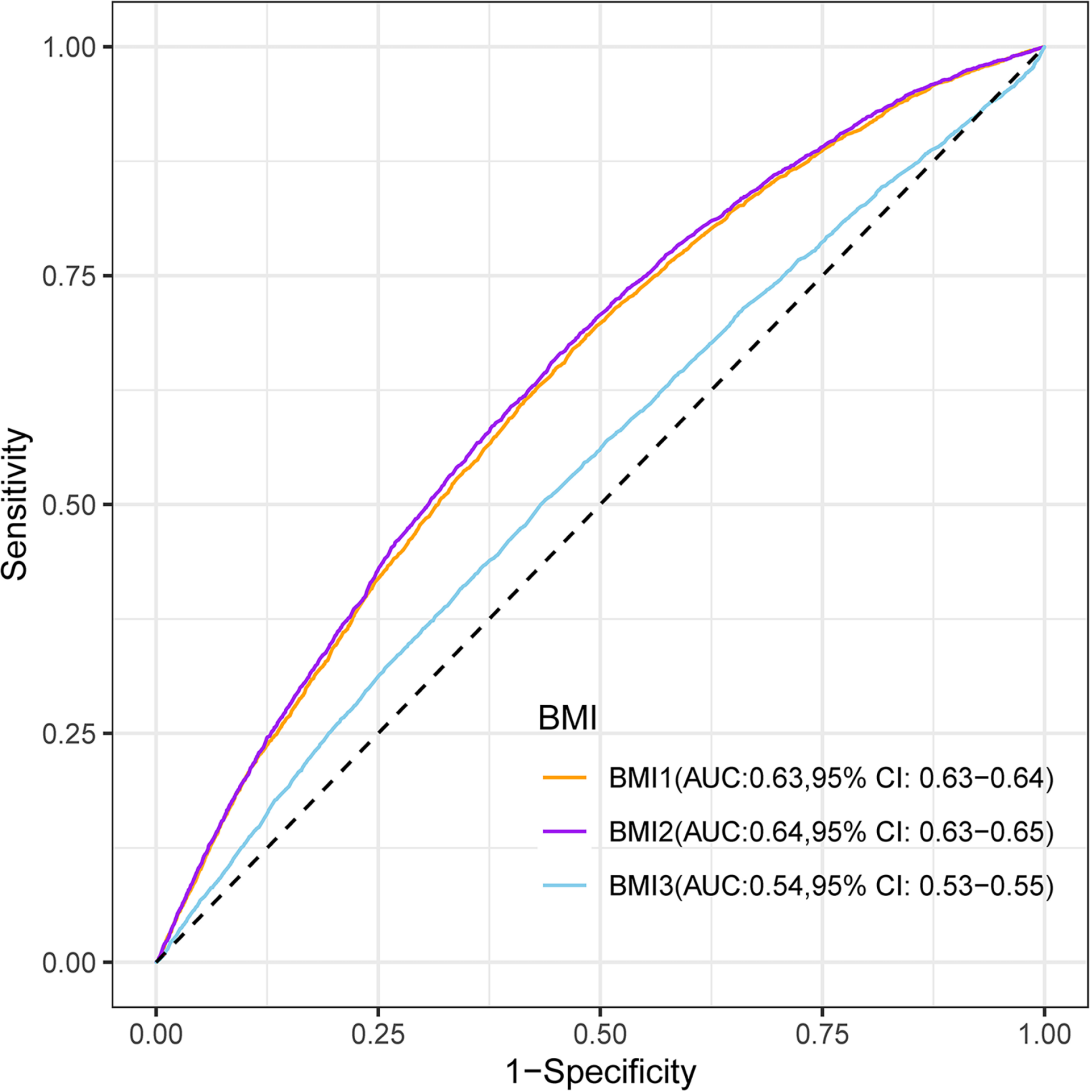
Receiver-operating characteristic curves for the prediction of macrosomia by maternal pre-pregnancy BMI (BMI1), maternal first-trimester BMI (BMI2), and paternal pre-pregnancy BMI (BMI3). BMI, body mass index; AUC, area under the receiver-operating characteristic curve; CI, confidence intervals.

Maternal first-trimester BMI showed better discrimination of macrosomia than maternal pre-pregnancy BMI (AUC: 0.64 versus 0.63; Delong test: *P* < 0.001) and paternal pre-pregnancy BMI (AUC: 0.64 versus 0.54; Delong test: *P* < 0.001) (**Figure 2**).

### The Relationship Between Maternal First-Trimester BMI and Macrosomia

The results of univariate analyses are given in **Table S2**. The results of non-adjusted and adjusted multivariate logistic regression models are shown in **Table 2**. In the crude model, maternal first-trimester low BMI showed protective effect against macrosomia (OR = 0.36, 95% CI: 0.31–0.42, *P* < 0.001). In minimally adjusted model (adjusted maternal age, parental ethnicity, parental educational level, parental occupational physical activity, and maternal income), the protective effect did not have obvious changes (aOR = 0.37, 95% CI: 0.32–0.43, *P* < 0.001). Adjusting for all the confounders (minimally adjusted model plus parental smoking, maternal secondhand smoke exposure, parental drinking before or during pregnancy, parity, mode of conception, folic acid and multivitamin supplementation, preterm birth, fetal sex, season of delivery, GDM/GDM history, hypertension/hypertension history, and thyroid disease/thyroid disease history) of this study also did not substantially change the protective effect (aOR = 0.37, 95% CI: 0.32–0.43).

**Table 2:** Relationship between maternal first-trimester BMI and macrosomia in different models.

Exposure	Crude model OR (95% CI)	Minimally adjusted model aOR (95% CI)	Fully adjusted model aOR (95% CI)
Maternal first-trimester BMI (continuous)	1.82 (1.76–1.89)	1.80 (1.73–1.87)	1.82 (1.75–1.89)
Maternal first-trimester BMI (category)			
Normal (BMI 18.5–24.9 kg/m^2^)	Ref	Ref	Ref
Low (BMI < 18.5 kg/m^2^)	0.36 (0.31–0.42)	0.37 (0.32–0.43)	0.37 (0.32–0.43)
Overweight (BMI 25.0–29.9 kg/m^2^)	1.97 (1.84–2.10)	1.92 (1.79–2.06)	1.93 (1.80–2.07)
Obesity (BMI ≥ 30.0 kg/m^2^)	2.39 (2.10–2.73)	2.32 (2.03–2.65)	2.37 (2.07–2.71)
*P* for trend	<0.001	<0.001	<0.001

Crude model: adjusted for none. Minimally adjusted model: adjusted for maternal age, parental ethnicity, parental educational level, parental occupational physical activity, and maternal income. Fully adjusted model: adjusted for minimally adjusted model plus parental smoking, maternal secondhand smoke exposure, parental drinking before or during pregnancy, parity, mode of conception, folic acid and multivitamin supplementation, preterm birth, fetal sex, season of delivery, GDM/GDM history, hypertension/hypertension history, and thyroid disease/thyroid disease history.

CI, confidence intervals; OR, odds ratios; aOR, adjusted odds ratios; Ref, reference.

### Stratified Analyses

**Figure 3:**
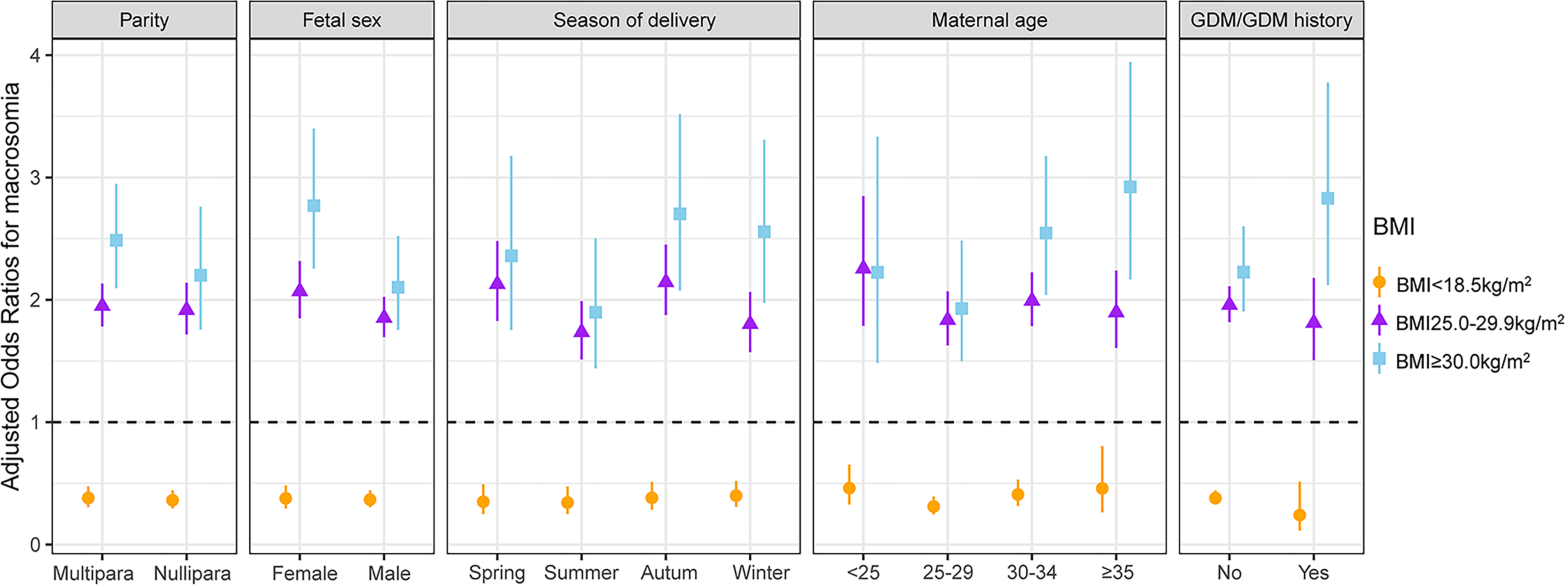
Interactive analyses of the association between maternal first-trimester BMI and macrosomia (each stratification adjusted for all the factors except for the stratification factor itself). All the factors included maternal age, parental ethnicity, parental educational level, parental occupational physical activity, maternal income, parental smoking, maternal secondhand smoke exposure, parental drinking before or during pregnancy, parity, mode of conception, folic acid and multivitamin supplementation, preterm birth, fetal sex, season of delivery, GDM/GDM history, hypertension/hypertension history, and thyroid disease/thyroid disease history. BMI, body mass index; GDM, gestational diabetes mellitus.

Stratified and interactive analyses showed that maternal first-trimester low BMI was inversely associated with macrosomia, irrespective of parity, fetal sex, season of delivery, maternal age, and GDM/GDM history status (**Figure 3**). As shown in **Table S3**, the protective effect of maternal first-trimester low BMI against macrosomia was significantly modified by fetal sex (interaction test *P* = 0.040), whereas parity, season of delivery, maternal age, and GDM/GDM history status could not modify the protective effect of the first-trimester low BMI against macrosomia (*P* for interaction = 0.927, 0.309, 0.282, and 0.169, respectively).

### Combined Effect of GDM/GDM History Status and Parity in Different Maternal Age Groups

**Figure 4:**
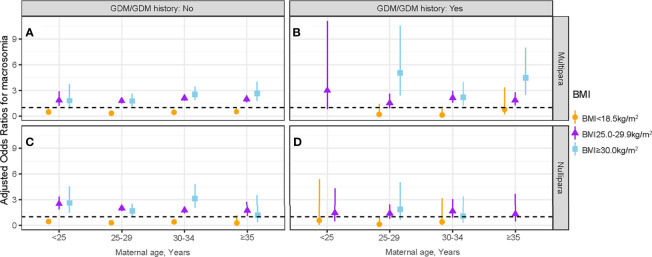
Association between maternal first-trimester BMI and macrosomia stratified by GDM/GDM history status, parity, and maternal age. The association between maternal first-trimester BMI and macrosomia in multipara without GDM/GDM history **(A)**, multipara with GDM/GDM history **(B)**, nullipara without GDM/GDM history **(C)** and nullipara with GDM/GDM history **(D)**. BMI, body mass index; GDM, gestational diabetes mellitus.

The interactive effects between maternal age and maternal first-trimester low BMI on macrosomia stratified by parity and GDM/GDM history status are presented in **Figure 4** and **Table S4**. Pregnant women without GDM/GDM history in the 25- to 29-year-old group were more benefited from a low BMI (<18.5 kg/m^2^) than other maternal age groups, irrespective of parity (multipara: aOR = 0.32, 95% CI: 0.22–0.47; nullipara: aOR = 0.32, 95% CI: 0.24–0.43) (**Figures 4A, C** and **Table S4**). In multipara with GDM/GDM history, the protective effect of maternal first-trimester low BMI was only observed in the 30- to 34-year-old group (aOR = 0.12, 95% CI: 0.02–0.86) (**Figure 4B**). No protective effect was observed in nullipara with GDM/GDM history (**Figure 4D**).

### Combined Effect of GDM/GDM History Status and Fetal Sex in Different Season of Delivery Groups

**Figure 5:**
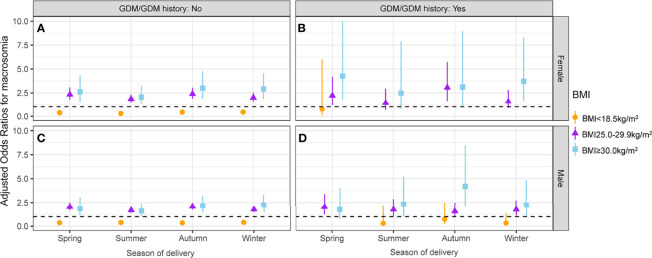
Association between maternal first-trimester BMI and macrosomia stratified by GDM/GDM history status, fetal sex, and season of delivery. The association between maternal first-trimester BMI and macrosomia in female fetuses whose mothers without GDM/GDM history **(A)**, female fetuses whose mothers with GDM/GDM history **(B)**, male fetuses whose mothers without GDM/GDM history **(C)** and male fetuses whose mothers with GDM/GDM history **(D)**. BMI, body mass index; GDM, gestational diabetes mellitus.

The interactive effects between season of delivery and first-trimester low BMI on macrosomia stratified by GDM/GDM history status and fetal sex are presented in **Figure 5** and **Table S5**. In pregnant women without GDM/GDM history, maternal first-trimester low BMI was inversely associated with macrosomia across all seasons of delivery groups, irrespective of fetal sex. For pregnant women without GDM/GDM history, the protective effect of maternal first-trimester low BMI (<18.5 kg/m^2^) against macrosomia was the weakest in infants born in winter, irrespective of fetal sex (female: aOR = 0.45, 95% CI: 0.29–0.69; male: aOR = 0.39, 95% CI: 0.28–0.55). The protective effect of maternal first-trimester low BMI against macrosomia was the strongest in female infants born in summer (aOR = 0.30, 95% CI: 0.17–0.52) and in male infants born in spring and autumn (spring: aOR = 0.35, 95% CI: 0.23–0.55; autumn: aOR = 0.35, 95% CI: 0.24–0.52) (**Figures 5A, C** and **Table S5**). No protective effect was observed in pregnant women with GDM/GDM history (**Figures 5B, D** **)**.

## Discussion

### Main Findings

In this 10-year cross-sectional study, maternal first-trimester BMI showed better prediction performance for fetal macrosomia compared with parental pre-pregnancy BMI. Maternal first-trimester low BMI is inversely associated with macrosomia, irrespective of parity, fetal sex, season of delivery, maternal age, and GDM/GDM history status. The protective effect of maternal first-trimester low BMI against macrosomia was most pronounced among 25- to 29-year-old pregnant women without GDM/GDM history and was only found among 30- to 34-year-old multipara with GDM/GDM history. The protective effect of maternal first-trimester low BMI against macrosomia was the weakest in winter of delivery among pregnant women without GDM/GDM history.

### Comparison With Previous Studies

To the best of our knowledge, the effect of maternal first-trimester BMI on macrosomia based on comprehensive information has seldom been investigated. Previous studies reported that maternal pre-pregnancy and first-trimester high BMI were independent risk factors for macrosomia (23, 24). The effect of maternal pre-pregnancy low BMI on macrosomia was less studied and the results were inconclusive. An updated meta-analysis, involving 1,392,799 pregnant women, reported that low pre-pregnancy BMI was a significant protective factor for the development of macrosomia (16), whereas such protective effect (OR = 0.75, 95% CI: 0.17–3.24) was not found in a cohort study (15).

Mounting evidence has demonstrated that the risk of macrosomia increases with parity (25, 26). Koyanagi et al. (27) performed a multicenter study based on 276,436 singleton livebirths or fresh stillbirths and demonstrated that higher parity was associated with a significantly increased risk of macrosomia. One possible explanation is that fetuses of nullipara were more likely to be exposed to a different maternal immune environment which might restrict the fetal growth in the uterine (28). Another possible explanation is that higher parity has been reported to be strictly related to GDM, which played an important role in the development of macrosomia (29). Schwartz et al. (13) found that multipara had a higher risk of GDM compared with nullipara (73% versus 40%, *P* < 0.001) based on a meta-analysis of 19,053 singleton participants. GDM increased the risk of macrosomia more than twice compared with non-GDM mothers (95% CI: 1.56–2.71; *P* < 0.001) (30).

The increased risk of fetal macrosomia in GDM mothers is mainly due the following reason: maternal hyperglycemia drives a higher amount of circulating glucose to pass through the placenta and reach the fetal circulation, whereas the maternal-derived or exogenous insulin could not pass the placenta (31). Meanwhile, higher glucose in fetal circulation results in increased fetal insulin secretion (32). Thus, the combinative effect of extra glucose and insulin leads to the increased accumulation of adipose tissue and protein in the fetuses, resulting in fetal accelerated growth and macrosomia (33, 34).

Pereda et al. (29) conducted a cross-sectional study involving 42,663 pregnant women and reported that male fetuses were associated with increased risk of macrosomia (aOR = 1.89, 95% CI: 1.72–2.08), and such association was also found in our study (OR = 1.69, 95% CI: 1.60–1.78) (**Table S2**). We found that for pregnant women without GDM/GDM history, the protective effect of maternal first-trimester low BMI against macrosomia was the strongest in female infants born in summer and in male infants born in spring and autumn. These findings were similar to those of Wu et al. (14) who reported that compared with infants born in spring, the risk of developing macrosomia for infants born in summer and autumn was low (summer: aOR = 0.85, 95% CI: 0.75–0.98; autumn: aOR = 0.87, 95% CI: 0.77–0.99). The protective effect of maternal first-trimester low BMI against macrosomia was the weakest in winter. The possible explanation is that maternal lower exposure to sunshine and reduced level of activity in winter could cause a lower level of maternal serum 25-hydroxyvitamin D, and 25-hydroxyvitamin D assists with a Th2 phenotype in the immunology of pregnancy (35).

### Strengths and Limitations

The current study has several strengths. First, our study has a large sample size of 84,900 participants based on a 10-year cross-sectional study, which enabled us to identify the effect of maternal first-trimester BMI on macrosomia in subgroups with convincing statistical results. Second, detailed information of participants was recorded, allowing us to adjust for potential confounding factors as much as possible, including demographic characteristics, lifestyle behaviors, maternal previous/current diseases, and current pregnancy information of the study population. Third, the value of weight and height of pregnant women in the first trimester was accurately measured, which reduced the potential reporting bias.

However, we acknowledge that several limitations cannot be neglected. First, although we used strict statistical adjustments to minimize residual confounding based on the recommendation of the STROBE statement, we cannot rule out the possibility of some residual effects related to unknown factors. Female pregnancy is a constantly changing process, during which there are many confounding factors that lead to the occurrence of macrosomia. Second, our hospital information system does not offer weight gain during pregnancy, which has been reported to be related to macrosomia (36), and this may have led to biased estimates. Third, participants with missing data and outliers of key variables were excluded, which may have contributed to bias in our findings, but we believe our final conclusion is robust because of the low missing and outlier rate of 0.39% (360 of 92,406). Fourth, our study population was from a single center, restricting the generalizability of the findings, and replication of our findings in other large populations is warranted. Besides, the current study did not include maternal information of diet/life style that could help reduce the risk of GDM; this may influence our results and we will collect the related information and study it in the future.

## Conclusion

Maternal first-trimester BMI is a good marker for macrosomia. Maternal first-trimester low BMI is inversely associated with macrosomia, irrespective of parity, fetal sex, season of delivery, maternal age, and GDM/GDM history status. The protective effect of maternal first-trimester low BMI against macrosomia was most pronounced among 25- to 29-year-old pregnant women without GDM/GDM history and was only found among 30- to 34-year-old multipara with GDM/GDM history. Expected delivery date for pregnant women in winter is not advisable. Further studies are required to shed light on the underlying mechanisms contributing to the protective effect of maternal first-trimester low BMI against macrosomia.

## Data Availability Statement

The original contributions presented in the study are included in the article/**Supplementary Material**. Further inquiries can be directed to the corresponding authors.

## Ethics Statement

The studies involving human participants were reviewed and approved by the Ethical Committee of Beijing Obstetrics and Gynecology Hospital, Capital Medical University (No. IEC-C-03-V04-FJ2). The patients/participants provided their written informed consent to participate in this study.

## Author Contributions

CY had full access to all the data of the study and takes responsibility for the integrity and accuracy of the data analysis. Concept and design: CY, WY, YS, MZ, and QW. Acquisition, analysis, or interpretation of data: YS, MZ, RL, CY, WY, and QW. Drafting of the manuscript: YS and MZ. Critical revisions of the manuscript for important intellectual content: all authors. Statistical analysis: MZ and YS. Obtained funding: CY, WY, YS and MZ. Administrative, technical, and material support: YS, MZ, RL, JW, KY, CY, and WY. Supervision: CY, WY, and QW. All authors contributed to the article and approved the submitted version.

## Funding

This work was supported by the National Key Research and Development Program of China (No. 2016YFC1000101, No. 2019YFC1005100), China Postdoctoral Science Foundation (No. 2020TQ0207), and Postdoctoral Foundation provided by Beijing Obstetrics and Gynecology Hospital, Capital Medical University.

## Conflict of Interest

The authors declare that the research was conducted in the absence of any commercial or financial relationships that could be construed as a potential conflict of interest.

## Publisher’s Note

All claims expressed in this article are solely those of the authors and do not necessarily represent those of their affiliated organizations, or those of the publisher, the editors and the reviewers. Any product that may be evaluated in this article, or claim that may be made by its manufacturer, is not guaranteed or endorsed by the publisher.

## References

[1] Committee on Practice Bulletins—Obstetrics. Macrosomia: ACOG Practice Bulletin, Number 216. Obstet Gynecol (2020) 135(1):e18–35. doi: 10.1097/AOG.0000000000003606 31856124

[2] Agay-Shay K Rudolf M Rubin L Haklai Z Grotto I. Trends in Fetal Growth Between 2000 to 2014 in Singleton Live Births From Israel. Sci Rep (2018) 8(1):1089. doi: 10.1038/s41598-018-19396-w 29348415 PMC5773590

[3] Beta J Khan N Khalil A Fiolna M Ramadan G Akolekar R. Maternal and Neonatal Complications of Fetal Macrosomia: Systematic Review and Meta-Analysis. Ultrasound Obstet Gynecol (2019) 54(3):308–18. doi: 10.1002/uog.20279 30938004

[4] Dyck RF Karunanayake C Pahwa P Osgood ND. The Hefty Fetal Phenotype Hypothesis Revisited: High Birth Weight, Type 2 Diabetes and Gestational Diabetes in a Saskatchewan Cohort of First Nations and Non-First Nations Women. J Dev Orig Health Dis (2019) 10(1):48–54. doi: 10.1017/S2040174417000988 29271332

[5] Black MH Sacks DA Xiang AH Lawrence JM. The Relative Contribution of Prepregnancy Overweight and Obesity, Gestational Weight Gain, and IADPSG-Defined Gestational Diabetes Mellitus to Fetal Overgrowth. Diabetes Care (2013) 36(1):56–62. doi: 10.2337/dc12-0741 22891256 PMC3526206

[6] Maheux-Lacroix S Li F Bujold E Nesbitt-Hawes E Deans R Abbott J. Cesarean Scar Pregnancies: A Systematic Review of Treatment Options. J Minim Invasive Gynecol (2017) 24(6):915–25. doi: 10.1016/j.jmig.2017.05.019 28599886

[7] Goldstein RF Abell SK Ranasinha S Misso M Boyle JA Black MH. Association of Gestational Weight Gain With Maternal and Infant Outcomes: A Systematic Review and Meta-Analysis. JAMA (2017) 317(21):2207–25. doi: 10.1001/jama.2017.3635 PMC5815056 28586887

[8] Goldstein RF Abell SK Ranasinha S Misso ML Boyle JA Harrison CL. Gestational Weight Gain Across Continents and Ethnicity: Systematic Review and Meta-Analysis of Maternal and Infant Outcomes in More Than One Million Women. BMC Med (2018) 16(1):153. doi: 10.1186/s12916-018-1128-1 30165842 PMC6117916

[9] Yang W Liu J Li J Liu J Liu H Wang Y. Interactive Effects of Prepregnancy Overweight and Gestational Diabetes on Macrosomia and Large for Gestational Age: A Population-Based Prospective Cohort in Tianjin, China. Diabetes Res Clin Pract (2019) 154:82–9. doi: 10.1016/j.diabres.2019.06.014 31271809

[10] Liu Y Guo F Zhou Y Yang X Zhang Y Fan J. The Interactive Effect of Prepregnancy Overweight/Obesity and Isolated Maternal Hypothyroxinemia on Macrosomia. J Clin Endocrinol Metab (2021) 106(7):e2639–46. doi: 10.1210/clinem/dgab171 33720320

[11] Dai RX He XJ Hu CL. The Association Between Advanced Maternal Age and Macrosomia: A Meta-Analysis. Child Obes (2019) 15(3):149–55. doi: 10.1089/chi.2018.0258 30730213

[12] Rao J Fan D Wu S Lin D Zhang H Ye S. Trend and Risk Factors of Low Birth Weight and Macrosomia in South China, 2005-2017: A Retrospective Observational Study. Sci Rep (2018) 8(1):3393. doi: 10.1038/s41598-018-21771-6 29467433 PMC5821827

[13] Schwartz N Nachum Z Green MS. The Prevalence of Gestational Diabetes Mellitus Recurrence–Effect of Ethnicity and Parity: A Metaanalysis. Am J Obstet Gynecol (2015) 213(3):310–7. doi: 10.1016/j.ajog.2015.03.011 25757637

[14] Wu L Ding Y Rui XL Mao CP. Seasonal Variations in Birth Weight in Suzhou Industrial Park. BioMed Environ Sci (2016) 29(10):758–61. doi: 10.3967/bes2016.101 27927276

[15] Liang CC Chao M Chang SD Chiu SY. Impact of Prepregnancy Body Mass Index on Pregnancy Outcomes, Incidence of Urinary Incontinence and Quality of Life During Pregnancy - An Observational Cohort Study. BioMed J (2020) 43(6):476–83. doi: 10.1016/j.bj.2019.11.001 PMC7804172 33246799

[16] Liu P Xu L Wang Y Zhang Y Du Y Sun Y. Association Between Perinatal Outcomes and Maternal Pre-Pregnancy Body Mass Index. Obes Rev (2016) 17(11):1091–102. doi: 10.1111/obr.12455 27536879

[17] Hunt K Wyke S Gray CM Anderson AS Brady A Bunn C. A Gender-Sensitised Weight Loss and Healthy Living Programme for Overweight and Obese Men Delivered by Scottish Premier League Football Clubs (FFIT): A Pragmatic Randomised Controlled Trial. Lancet (2014) 383(9924):1211–21. doi: 10.1016/S0140-6736(13)62420-4 PMC4524002 24457205

[18] Bragg F Tang K Guo Y Iona A Du H Holmes MV. Associations of General and Central Adiposity With Incident Diabetes in Chinese Men and Women. Diabetes Care (2018) 41(3):494–502. doi: 10.2337/dc17-1852 29298802 PMC6548563

[19] Dipasquale V Ventimiglia M Gramaglia S Parma B Funari C Selicorni A. Caregiver Social Status and Health-Related Quality of Life in Neurologically Impaired Children on Home Enteral Nutrition. Nutrients (2021) 13(6):1928. doi: 10.3390/nu13061928 34199721 PMC8228116

[20] Liu Y Zhu Y Jia W Sun D Zhao L Zhang C. Association Between Lipid Profiles and Presence of Carotid Plaque. Sci Rep (2019) 9(1):18011. doi: 10.1038/s41598-019-54285-w 31784590 PMC6884522

[21] Carroll X Liang X Zhang W Zhang W Liu G Turner N. Socioeconomic, Environmental and Lifestyle Factors Associated With Gestational Diabetes Mellitus: A Matched Case-Control Study in Beijing, China. Sci Rep (2018) 8(1):8103. doi: 10.1038/s41598-018-26412-6 29802340 PMC5970220

[22] Antoun E Kitaba NT Titcombe P Dalrymple KV Garratt ES Barton SJ. Maternal Dysglycaemia, Changes in the Infant’s Epigenome Modified With a Diet and Physical Activity Intervention in Pregnancy: Secondary Analysis of a Randomised Control Trial. PloS Med (2020) 17(11):e1003229. doi: 10.1371/journal.pmed.1003229 33151971 PMC7643947

[23] Sun Y Shen Z Zhan Y Wang Y Ma S Zhang S. Effects of Pre-Pregnancy Body Mass Index and Gestational Weight Gain on Maternal and Infant Complications. BMC Pregnancy Childbirth (2020) 20(1):390. doi: 10.1186/s12884-020-03071-y 32631269 PMC7336408

[24] Uchinuma H Tsuchiya K Sekine T Horiuchi S Kushima M Otawa S. Gestational Body Weight Gain and Risk of Low Birth Weight or Macrosomia in Women of Japan: A Nationwide Cohort Study. Int J Obes (Lond) (2021) 45(12):2666–74. doi: 10.1038/s41366-021-00947-7 PMC8606312 34465856

[25] Kim SY Kotelchuck M Wilson HG Diop H Shapiro-Mendoza CK England LJ. Prevalence of Adverse Pregnancy Outcomes, by Maternal Diabetes Status at First and Second Deliveries, Massachusetts, 1998-2007. Prev Chronic Dis (2015) 12:E218. doi: 10.5888/pcd12.150362 26652218 PMC4676277

[26] Lei F Zhang L Shen Y Zhao Y Kang Y Qu P. Association Between Parity and Macrosomia in Shaanxi Province of Northwest China. Ital J Pediatr (2020) 46(1):24. doi: 10.1186/s13052-020-0784-x 32070407 PMC7029605

[27] Koyanagi A Zhang J Dagvadorj A Hirayama F Shibuya K Souza JP. Macrosomia in 23 Developing Countries: An Analysis of a Multicountry, Facility-Based, Cross-Sectional Survey. Lancet (2013) 381(9865):476–83. doi: 10.1016/S0140-6736(12)61605-5 23290494

[28] Li G Xing Y Wang G Zhang J Wu Q Ni W. Differential Effect of Pre-Pregnancy Low BMI on Fetal Macrosomia: A Population-Based Cohort Study. BMC Med (2021) 19(1):175. doi: 10.1186/s12916-021-02046-w 34344359 PMC8335988

[29] Pereda J Bove I Pineyro MM. Excessive Maternal Weight and Diabetes Are Risk Factors for Macrosomia: A Cross-Sectional Study of 42,663 Pregnancies in Uruguay. Front Endocrinol (Lausanne) (2020) 11:588443. doi: 10.3389/fendo.2020.588443 33224106 PMC7669744

[30] Agudelo-Espitia V Parra-Sosa BE Restrepo-Mesa SL. Factors Associated With Fetal Macrosomia. Rev Saude Publica (2019) 53:100. doi: 10.11606/s1518-8787.2019053001269 31800911 PMC6863107

[31] Kerenyi Z Tamas G Kivimaki M Peterfalvi A Madarasz E Bosnyak Z. Maternal Glycemia and Risk of Large-for-Gestational-Age Babies in a Population-Based Screening. Diabetes Care (2009) 32(12):2200–5. doi: 10.2337/dc09-1088 PMC2782977 19729526

[32] Qiao L Wattez JS Lim L Rozance PJ Hay WJ Shao J. Prolonged Prepregnant Maternal High-Fat Feeding Reduces Fetal and Neonatal Blood Glucose Concentrations by Enhancing Fetal Beta-Cell Development in C57BL/6 Mice. Diabetes (2019) 68(8):1604–13. doi: 10.2337/db18-1308 PMC6692812 31127056

[33] Baier LJ Muller YL Remedi MS Traurig M Piaggi P Wiessner G. ABCC8 R1420H Loss-Of-Function Variant in a Southwest American Indian Community: Association With Increased Birth Weight and Doubled Risk of Type 2 Diabetes. Diabetes (2015) 64(12):4322–32. doi: 10.2337/db15-0459 PMC4657583 26246406

[34] de Souza ML Caria C Santos PS Ruy CC Da SLN Moreira D. Modulatory Effect of Polyphenolic Compounds From the Mangrove Tree Rhizophora Mangle L. @ on Non-Alcoholic Fatty Liver Disease and Insulin Resistance in High-Fat Diet Obese Mice. Molecules (2018) 23(9):2114. doi: 10.3390/molecules23092114 PMC6225131 30135414

[35] Wen J Kang C Wang J Cui X Hong Q Wang X. Association of Maternal Serum 25-Hydroxyvitamin D Concentrations in Second and Third Trimester With Risk of Macrosomia. Sci Rep (2018) 8(1):6169. doi: 10.1038/s41598-018-24534-5 29670182 PMC5906563

[36] McDowell M Cain MA Brumley J. Excessive Gestational Weight Gain. J Midwifery Womens Health (2019) 64(1):46–54. doi: 10.1111/jmwh.12927 30548447

