# Supplementary material for: Protective Effect of Maternal First-Trimester Low Body Mass Index Against Macrosomia: A 10-Year Cross-Sectional Study
Source: Front Endocrinol (Lausanne). 2022 Feb 10;13:805636. doi: 10.3389/fendo.2022.805636 (PMC8866317; doi:10.3389/fendo.2022.805636)
Supplement: Supplementary file 1 [file Table_1.docx]

**TABLE S1 |** All characteristics of participants

| **Characteristics** | **Maternal first-trimester body mass index** | | | | **P** |
| --- | --- | --- | --- | --- | --- |
|  | **Normal** | **Low** | **Overweight** | **Obesity** |  |
| **Maternal age (years)** | 30.25 (4.16) | 28.53 (3.85) | 30.99 (4.48) | 30.55 (4.68) | <0.001 |
| **Macrosomia, n (%)** |  |  |  |  | <0.001 |
| No | 58514 (94.35) | 8640 (97.88) | 10661 (89.47) | 1870 (87.46) |  |
| Yes | 3505 (5.65) | 187 (2.12) | 1255 (10.53) | 268 (12.54) |  |
| **Maternal ethnicity, n (%)** |  |  |  |  | 0.482 |
| Han | 58209 (93.86) | 8267 (93.66) | 11159 (93.65) | 1992 (93.17) |  |
| Minority | 3810 (6.14) | 560 (6.34) | 757 (6.35) | 146 (6.83) |  |
| **Paternal ethnicity, n (%)** |  |  |  |  | 0.228 |
| Han | 58896 (94.96) | 8360 (94.71) | 11265 (94.54) | 2032 (95.04) |  |
| Minority | 3123 (5.04) | 467 (5.29) | 651 (5.46) | 106 (4.96) |  |
| **Maternal educational level, n (%)** |  |  |  |  | <0.001 |
| College/university | 26281 (42.38) | 3606 (40.85) | 4509 (37.84) | 626 (29.28) |  |
| High school or below | 28608 (46.13) | 4388 (49.71) | 6551 (54.98) | 1439 (67.31) |  |
| Postgraduate | 7126 (11.49) | 833 (9.44) | 856 (7.18) | 73 (3.41) |  |
| **Paternal educational level, n (%)** |  |  |  |  | <0.001 |
| College/university | 26723 (43.09) | 3617 (40.98) | 4232 (35.52) | 572 (26.75) |  |
| High school or below | 28064 (45.25) | 4298 (48.69) | 6791 (56.99) | 1488 (69.60) |  |
| Postgraduate | 7231 (11.66) | 912 (10.33) | 893 (7.49) | 78 (3.65) |  |
| **Maternal occupational physical activity, n (%)** |  |  |  |  | <0.001 |
| Moderate | 15592 (25.14) | 2173 (24.62) | 2760 (23.16) | 434 (20.30) |  |
| Active | 31967 (51.55) | 4325 (49.00) | 5769 (48.41) | 977 (45.70) |  |
| Light | 14459 (23.31) | 2329 (26.38) | 3387 (28.43) | 727 (34.00) |  |
| **Paternal occupational physical activity, n (%)** |  |  |  |  | <0.001 |
| Moderate | 12714 (20.50) | 1716 (19.44) | 2192 (18.39) | 363 (16.98) |  |
| Active | 33563 (54.12) | 4493 (50.90) | 6327 (53.10) | 1063 (49.72) |  |
| Light | 15742 (25.38) | 2618 (29.66) | 3397 (28.51) | 712 (33.30) |  |
| **Maternal income (Yuan), n (%)** |  |  |  |  | <0.001 |
| <50000 | 17436 (28.12) | 2832 (32.08) | 3982 (33.42) | 902 (42.19) |  |
| 50000-100000 | 24642 (39.73) | 3437 (38.94) | 4845 (40.66) | 814 (38.07) |  |
| >100000 | 19940 (32.15) | 2558 (28.98) | 3089 (25.92) | 422 (19.74) |  |
| **Parity, n (%)** |  |  |  |  | <0.001 |
| Multipara | 31648 (51.03) | 3684 (41.74) | 7046 (59.13) | 1288 (60.24) |  |
| Nullipara | 30371 (48.97) | 5143 (58.26) | 4870 (40.87) | 850 (39.76) |  |
| **Maternal smoking before or during pregnancy, n (%)** |  |  |  |  | <0.001 |
| No | 60697 (97.87) | 8594 (97.36) | 11606 (97.40) | 2039 (95.37) |  |
| Yes | 1321 (2.13) | 233 (2.64) | 310 (2.60) | 99 (4.63) |  |
| **Paternal smoking before or during pregnancy, n (%)** |  |  |  |  | <0.001 |
| No | 37558 (60.56) | 5079 (57.54) | 6896 (57.88) | 1095 (51.22) |  |
| Yes | 24460 (39.44) | 3748 (42.46) | 5019 (42.12) | 1043 (48.78) |  |
| **Maternal secondhand smoke exposure before or during pregnancy, n (%)** |  |  |  |  | 0.097 |
| No | 54540 (88.11) | 7732 (87.88) | 10439 (87.83) | 1842 (86.36) |  |
| Yes | 7357 (11.89) | 1066 (12.12) | 1447 (12.17) | 291 (13.64) |  |
| **Maternal drinking before or during pregnancy, n (%)** |  |  |  |  | 0.029 |
| No | 59925 (96.63) | 8554 (96.91) | 11568 (97.09) | 2057 (96.21) |  |
| Yes | 2093 (3.37) | 273 (3.09) | 347 (2.91) | 81 (3.79) |  |
| **Paternal drinking before or during pregnancy, n (%)** |  |  |  |  | 0.021 |
| No | 43364 (69.92) | 6129 (69.43) | 8346 (70.05) | 1428 (66.79) |  |
| Yes | 18652 (30.08) | 2698 (30.57) | 3569 (29.95) | 710 (33.21) |  |
| **Mode of conception, n (%)** |  |  |  |  | <0.001 |
| Natural conception | 59712 (96.28) | 8589 (97.30) | 11310 (94.91) | 2028 (94.86) |  |
| Assisted reproduction | 2307 (3.72) | 238 (2.740) | 606 (5.09) | 110 (5.14) |  |
| **Folic acid supplementation, n (%)** |  |  |  |  | <0.001 |
| No | 4507 (7.27) | 663 (7.51) | 855 (7.18) | 184 (8.61) |  |
| Yes | 57512 (92.73) | 8164 (92.49) | 11061 (92.82) | 1954 (91.39) |  |
| **Multivitamin supplementation, n (%)** |  |  |  |  | <0.001 |
| No | 22212 (35.81) | 3473 (39.35) | 4267 (35.81) | 859 (40.18) |  |
| Yes | 39807 (64.19) | 5354 (60.65) | 7649 (64.19) | 1279 (59.82) |  |
| **Preterm birth, n (%)** |  |  |  |  | <0.001 |
| No | 60407 (97.41) | 8632 (97.79) | 11506 (96.59) | 2042 (95.51) |  |
| Yes | 1605 (2.59) | 195 (2.21) | 406 (3.41) | 96 (4.49) |  |
| **Fetal sex, n (%)** |  |  |  |  | 0.007 |
| Male | 32408 (52.25) | 4452 (50.44) | 6271 (52.63) | 1098 (51.36) |  |
| Female | 29611 (47.75) | 4375 (49.56) | 5645 (47.37) | 1040 (48.64) |  |
| **Season of delivery, n (%)** |  |  |  |  | <0.001 |
| Spring | 11637 (18.76) | 1657 (18.77) | 2282 (19.15) | 433 (20.25) |  |
| Summer | 16907 (27.26) | 2102 (23.82) | 3400 (28.53) | 611 (28.58) |  |
| Autumn | 17385 (28.03) | 2515 (28.49) | 3303 (27.72) | 571 (26.71) |  |
| Winter | 16090 (25.95) | 2553 (28.92) | 2931 (24.60) | 523 (24.46) |  |
| **GDM/GDM history, n (%)** |  |  |  |  | <0.001 |
| No | 56262 (90.72) | 8381 (94.95) | 9955 (83.54) | 1695 (79.28) |  |
| Yes | 5757 (9.28) | 446 (5.05) | 1961 (16.46) | 443 (20.72) |  |
| **Gestational hypertension/ Gestational hypertension history, n (%)** |  |  |  |  | <0.001 |
| No | 56993 (91.90) | 8371 (94.83) | 10204 (85.63) | 1668 (78.02) |  |
| Yes | 5026 (8.10) | 456 (5.17) | 1712 (14.37) | 470 (21.98) |  |
| **Thyroid disease/Thyroid disease history, n (%)** |  |  |  |  | <0.001 |
| No | 56397 (90.94) | 8113 (91.91) | 10730 (90.05) | 1924 (89.99) |  |
| Yes | 5622 (9.06) | 714 (8.09) | 1186 (9.95) | 214 (10.01) |  |

*Data are given as mean (SD), or n (%). Abbreviations: GDM, gestational diabetes mellitus.*
