# Supplementary material for: Protective Effect of Maternal First-Trimester Low Body Mass Index Against Macrosomia: A 10-Year Cross-Sectional Study
Source: Front Endocrinol (Lausanne). 2022 Feb 10;13:805636. doi: 10.3389/fendo.2022.805636 (PMC8866317; doi:10.3389/fendo.2022.805636)
Supplement: Supplementary file 2 [file Table_2.docx]

**Table S2** **|** The results of univariate analysis

| **Characteristics** | **OR (95% CI)** | **P** |
| --- | --- | --- |
| **Maternal age** | 1.12 (1.08-1.16) | 0.001 |
| **Maternal pre-pregnancy BMI** | 1.76 (1.70-1.83) | 0.001 |
| **Maternal first-trimester BMI** | 1.82 (1.76-1.89) | 0.001 |
| **Paternal pre-pregnancy BMI** | 1.20 (1.15-1.25) | 0.001 |
| **Maternal ethnicity** |  |  |
| Han | Ref |  |
| Minority | 0.81 (0.71-0.92) | 0.001 |
| **Maternal** **educational level** |  |  |
| College/university | Ref |  |
| High school or below | 1.08 (1.02-1.15) |  |
| Postgraduate | 0.88 (0.80-0.98) | 0.001 |
| **Parity** |  |  |
| Multiparous | Ref |  |
| Nulliparous | 0.80 (0.76-0.85) | 0.001 |
| **Preterm birth** |  |  |
| No | Ref |  |
| Yes | 0.10 (0.06-0.16) | 0.001 |
| **Fetal sex** |  |  |
| Female | Ref |  |
| Male | 1.69 (1.60-1.78) | 0.001 |
| **Season of delivery** |  |  |
| Spring | Ref |  |
| Summer | 0.89 (0.82-0.96) |  |
| Autumn | 0.88 (0.81-0.95) |  |
| Winter | 1.01 (0.94-1.09) | 0.001 |
| **GDM/GDM history** |  |  |
| No | Ref |  |
| Yes | 1.23 (1.13-1.34) | 0.001 |
| **Gestational hypertension/ Gestational hypertension history** |  |  |
| No | Ref |  |
| Yes | 1.20 (1.10-1.31) | 0.001 |
| **Maternal drinking before/during pregnancy** |  |  |
| No | Ref |  |
| Yes | 1.23 (1.07-1.42) | 0.006 |
| **Maternal smoking before/during pregnancy** |  |  |
| No | Ref |  |
| Yes | 1.23 (1.04-1.46) | 0.019 |
| **Maternal occupational physical activity** |  |  |
| Moderate | Ref |  |
| Active | 1.03 (0.96-1.11) |  |
| Light | 1.10 (1.02-1.19) | 0.054 |
| **Maternal income** |  |  |
| <50,000 | Ref |  |
| 50,000-100,000 | 0.97 (0.91-1.04) |  |
| >100,000 | 0.97 (0.90-1.04) | 0.600 |
| **Maternal secondhand smoke exposure before/during pregnancy** |  |  |
| No | Ref |  |
| Yes | 0.99 (0.91-1.08) | 0.838 |
| **Mode of conception** |  |  |
| Natural conceived | Ref |  |
| Artificial insemination | 1.01 (0.68-1.51) |  |
| Test-tube baby | 1.08 (0.93-1.26) | 0.618 |
| **Folic acid supplementation** |  |  |
| No | Ref |  |
| First-trimester | 1.00 (0.89-1.12) |  |
| Pre-pregnancy | 0.99 (0.88-1.11) | 0.960 |
| **Multivitamin supplementation** |  |  |
| No | Ref |  |
| First-trimester | 1.04 (0.97-1.11) |  |
| Pre-pregnancy | 1.01 (0.94-1.09) | 0.495 |
| **Thyroid disease/Thyroid disease history** |  |  |
| No | Ref |  |
| Yes | 0.93 (0.84-1.02) | 0.130 |

*Abbreviations: BMI, body mass index; GDM, gestational diabetes mellitus; CI, confidence intervals; OR, odds ratios; Ref, reference.*
