# Supplementary material for: Protective Effect of Maternal First-Trimester Low Body Mass Index Against Macrosomia: A 10-Year Cross-Sectional Study
Source: Front Endocrinol (Lausanne). 2022 Feb 10;13:805636. doi: 10.3389/fendo.2022.805636 (PMC8866317; doi:10.3389/fendo.2022.805636)
Supplement: Supplementary file 3 [file Table_3.docx]

**Table S3 |** Interactive analyses of the association between maternal first-trimester low BMI and macrosomia

| **Characteristics** | **Strata** | **BMI** | **aOR (95% CI)** | **P for interaction** |
| --- | --- | --- | --- | --- |
| **Parity** | Nullipara | Low | 0.36 (0.30-0.44) | 0.927 |
|  | Nullipara | Overweight | 1.92 (1.72-2.14) |  |
|  | Nullipara | Obesity | 2.20 (1.76-2.76) |  |
|  | Multipara | Low | 0.38 (0.31-0.47) |  |
|  | Multipara | Overweight | 1.95 (1.78-2.13) |  |
|  | Multipara | Obesity | 2.49 (2.10-2.95) |  |
| **Fetal sex** | Male | Low | 0.37 (0.30-0.44) | 0.040 |
|  | Male | Overweight | 1.85 (1.70-2.02) |  |
|  | Male | Obesity | 2.10 (1.75-2.52) |  |
|  | Female | Low | 0.38 (0.29-0.48) |  |
|  | Female | Overweight | 2.07 (1.85-2.32) |  |
|  | Female | Obesity | 2.77 (2.26-3.40) |  |
| **Season of delivery** | Spring | Low | 0.35 (0.25-0.49) | 0.309 |
|  | Spring | Overweight | 2.13 (1.83-2.48) |  |
|  | Spring | Obesity | 2.36 (1.75-3.17) |  |
|  | Summer | Low | 0.34 (0.25-0.47) |  |
|  | Summer | Overweight | 1.73 (1.51-1.99) |  |
|  | Summer | Obesity | 1.90 (1.44-2.50) |  |
|  | Autumn | Low | 0.38 (0.29-0.51) |  |
|  | Autumn | Overweight | 2.14 (1.88-2.45) |  |
|  | Autumn | Obesity | 2.70 (2.08-3.52) |  |
|  | Winter | Low | 0.40 (0.31-0.52) |  |
|  | Winter | Overweight | 1.80 (1.57-2.06) |  |
|  | Winter | Obesity | 2.56 (1.98-3.31) |  |
| **Maternal age** | <25 | Low | 0.46 (0.33-0.65) | 0.282 |
|  | <25 | Overweight | 2.26 (1.79-2.85) |  |
|  | <25 | Obesity | 2.22 (1.49-3.33) |  |
|  | 25-29 | Low | 0.31 (0.25-0.39) |  |
|  | 25-29 | Overweight | 1.83 (1.63-2.07) |  |
|  | 25-29 | Obesity | 1.93 (1.50-2.48) |  |
|  | 30-34 | Low | 0.41 (0.32-0.53) |  |
|  | 30-34 | Overweight | 1.99 (1.78-2.22) |  |
|  | 30-34 | Obesity | 2.54 (2.04-3.17) |  |
|  | ≥35 | Low | 0.46 (0.26-0.80) |  |
|  | ≥35 | Overweight | 1.90 (1.61-2.24) |  |
|  | ≥35 | Obesity | 2.92 (2.17-3.94) |  |
| **GDM/GDM history** | No | Low | 0.38 (0.33-0.44) | 0.169 |
|  | No | Overweight | 1.96 (1.82-2.11) |  |
|  | No | Obesity | 2.23 (1.91-2.60) |  |
|  | Yes | Low | 0.24 (0.11-0.51) |  |
|  | Yes | Overweight | 1.81 (1.51-2.18) |  |
|  | Yes | Obesity | 2.83 (2.12-3.78) |  |

*Abbreviations: BMI, body mass index; GDM, gestational diabetes mellitus; CI, confidence intervals; aOR, adjusted odds ratios; maternal first-trimester normal group is reference.*
