# Supplementary material for: Protective Effect of Maternal First-Trimester Low Body Mass Index Against Macrosomia: A 10-Year Cross-Sectional Study
Source: Front Endocrinol (Lausanne). 2022 Feb 10;13:805636. doi: 10.3389/fendo.2022.805636 (PMC8866317; doi:10.3389/fendo.2022.805636)
Supplement: Supplementary file 4 [file Table_4.docx]

**Table S4 |** Association between maternal first-trimester BMI and macrosomia stratified by GDM/GDM history status, parity, and maternal age

| **BMI** | **aOR (95% CI)** | **Maternal age** | **Parity** | **GDM/GDM history** |
| --- | --- | --- | --- | --- |
| Normal | Ref |  |  |  |
| Low | 0.46 (0.24-0.87) | <25 | Multipara | No |
| Overweight | 1.85 (1.19-2.88) | <25 | Multipara | No |
| Obesity | 1.79 (0.86-3.75) | <25 | Multipara | No |
| Normal | Ref |  |  |  |
| Low | 0.32 (0.22-0.47) | 25-29 | Multipara | No |
| Overweight | 1.76 (1.45-2.14) | 25-29 | Multipara | No |
| Obesity | 1.75 (1.17-2.63) | 25-29 | Multipara | No |
| Normal | Ref |  |  |  |
| Low | 0.43 (0.30-0.61) | 30-34 | Multipara | No |
| Overweight | 2.11 (1.82-2.43) | 30-34 | Multipara | No |
| Obesity | 2.55 (1.88-3.44) | 30-34 | Multipara | No |
| Normal | Ref |  |  |  |
| Low | 0.50 (0.26-0.99) | ≥35 | Multipara | No |
| Overweight | 1.98 (1.61-2.43) | ≥35 | Multipara | No |
| Obesity | 2.66 (1.77-4.00) | ≥35 | Multipara | No |
| Normal | Ref |  |  |  |
| Low | 0.47 (0.31-0.72) | <25 | Nullipara | No |
| Overweight | 2.52 (1.88-3.39) | <25 | Nullipara | No |
| Obesity | 2.63 (1.51-4.57) | <25 | Nullipara | No |
| Normal | Ref |  |  |  |
| Low | 0.32 (0.24-0.43) | 25-29 | Nullipara | No |
| Overweight | 2.01 (1.70-2.38) | 25-29 | Nullipara | No |
| Obesity | 1.68 (1.12-2.53) | 25-29 | Nullipara | No |
| Normal | Ref |  |  |  |
| Low | 0.42 (0.28-0.63) | 30-34 | Nullipara | No |
| Overweight | 1.76 (1.42-2.19) | 30-34 | Nullipara | No |
| Obesity | 3.16 (2.06-4.83) | 30-34 | Nullipara | No |
| Normal | Ref |  |  |  |
| Low | 0.28 (0.07-1.16) | ≥35 | Nullipara | No |
| Overweight | 1.75 (1.11-2.75) | ≥35 | Nullipara | No |
| Obesity | 1.20 (0.41-3.55) | ≥35 | Nullipara | No |
| Normal | Ref |  |  |  |
| Low | 0.00 (0.00-Inf) | <25 | Multipara | Yes |
| Overweight | 2.99 (0.81-11.08) | <25 | Multipara | Yes |
| Obesity | 2.04 (0.21-19.59) | <25 | Multipara | Yes |
| Normal | Ref |  |  |  |
| Low | 0.19 (0.03-1.40) | 25-29 | Multipara | Yes |
| Overweight | 1.53 (0.89-2.63) | 25-29 | Multipara | Yes |
| Obesity | 5.04 (2.41-10.52) | 25-29 | Multipara | Yes |
| Normal | Ref |  |  |  |
| Low | 0.12 (0.02-0.86) | 30-34 | Multipara | Yes |
| Overweight | 2.13 (1.54-2.93) | 30-34 | Multipara | Yes |
| Obesity | 2.20 (1.23-3.95) | 30-34 | Multipara | Yes |
| Normal | Ref |  |  |  |
| Low | 0.77 (0.18-3.34) | ≥35 | Multipara | Yes |
| Overweight | 1.86 (1.25-2.76) | ≥35 | Multipara | Yes |
| Obesity | 4.47 (2.50-7.97) | ≥35 | Multipara | Yes |
| Normal | Ref |  |  |  |
| Low | 0.59 (0.06-5.39) | <25 | Nullipara | Yes |
| Overweight | 1.46 (0.49-4.33) | <25 | Nullipara | Yes |
| Obesity | 3.32 (0.76-14.44) | <25 | Nullipara | Yes |
| Normal | Ref |  |  |  |
| Low | 0.14 (0.02-1.01) | 25-29 | Nullipara | Yes |
| Overweight | 1.40 (0.80-2.44) | 25-29 | Nullipara | Yes |
| Obesity | 1.87 (0.69-5.05) | 25-29 | Nullipara | Yes |
| Normal | Ref |  |  |  |
| Low | 0.42 (0.06-3.18) | 30-34 | Nullipara | Yes |
| Overweight | 1.66 (0.91-3.05) | 30-34 | Nullipara | Yes |
| Obesity | 1.09 (0.36-3.36) | 30-34 | Nullipara | Yes |
| Normal | Ref |  |  |  |
| Low | 0.00 (0.00-Inf) | ≥35 | Nullipara | Yes |
| Overweight | 1.33 (0.48-3.67) | ≥35 | Nullipara | Yes |
| Obesity | 4.51 (1.21-16.77) | ≥35 | Nullipara | Yes |

*Abbreviations: GDM, gestational diabetes mellitus; CI, confidence intervals; aOR, adjusted odds ratios; Ref, reference.*
