# Supplementary material for: Protective Effect of Maternal First-Trimester Low Body Mass Index Against Macrosomia: A 10-Year Cross-Sectional Study
Source: Front Endocrinol (Lausanne). 2022 Feb 10;13:805636. doi: 10.3389/fendo.2022.805636 (PMC8866317; doi:10.3389/fendo.2022.805636)
Supplement: Supplementary file 5 [file Table_5.docx]

**Table S5** **|** Association between maternal first-trimester BMI and macrosomia stratified by GDM/GDM history status, fetal sex, and season of delivery

| **BMI** | **aOR (95% CI)** | **Season of delivery** | **Fetal sex** | **GDM/GDM history** |
| --- | --- | --- | --- | --- |
| Normal | Ref |  |  |  |
| Low | 0.38 (0.21-0.67) | Spring | female | No |
| Overweight | 2.34 (1.79-3.06) | Spring | female | No |
| Obesity | 2.60 (1.56-4.33) | Spring | female | No |
| Normal | Ref |  |  |  |
| Low | 0.30 (0.17-0.52) | Summer | female | No |
| Overweight | 1.86 (1.48-2.33) | Summer | female | No |
| Obesity | 2.06 (1.30-3.26) | Summer | female | No |
| Normal | Ref |  |  |  |
| Low | 0.44 (0.27-0.71) | Autumn | female | No |
| Overweight | 2.40 (1.89-3.05) | Autumn | female | No |
| Obesity | 2.99 (1.88-4.76) | Autumn | female | No |
| Normal | Ref |  |  |  |
| Low | 0.45 (0.29-0.69) | Winter | female | No |
| Overweight | 2.00 (1.57-2.54) | Winter | female | No |
| Obesity | 2.91 (1.87-4.54) | Winter | female | No |
| Normal | Ref |  |  |  |
| Low | 0.35 (0.23-0.55) | Spring | male | No |
| Overweight | 2.04 (1.65-2.52) | Spring | male | No |
| Obesity | 1.86 (1.14-3.04) | Spring | male | No |
| Normal | Ref |  |  |  |
| Low | 0.38 (0.25-0.57) | Summer | male | No |
| Overweight | 1.69 (1.40-2.04) | Summer | male | No |
| Obesity | 1.59 (1.05-2.41) | Summer | male | No |
| Normal | Ref |  |  |  |
| Low | 0.35 (0.24-0.52) | Autumn | male | No |
| Overweight | 2.07 (1.73-2.48) | Autumn | male | No |
| Obesity | 2.16 (1.45-3.21) | Autumn | male | No |
| Normal | Ref |  |  |  |
| Low | 0.39 (0.28-0.55) | Winter | male | No |
| Overweight | 1.77 (1.46-2.14) | Winter | male | No |
| Obesity | 2.24 (1.51-3.31) | Winter | male | No |
| Normal | Ref |  |  |  |
| Low | 0.77 (0.10-6.00) | Spring | female | Yes |
| Overweight | 2.22 (1.18-4.17) | Spring | female | Yes |
| Obesity | 4.26 (1.81-10.01) | Spring | female | Yes |
| Normal | Ref |  |  |  |
| Low | 0.00 (0.00-Inf) | Summer | female | Yes |
| Overweight | 1.42 (0.69-2.93) | Summer | female | Yes |
| Obesity | 2.47 (0.78-7.89) | Summer | female | Yes |
| Normal | Ref |  |  |  |
| Low | 0.00 (0.00-Inf) | Autumn | female | Yes |
| Overweight | 3.06 (1.63-5.73) | Autumn | female | Yes |
| Obesity | 3.11 (1.08-8.96) | Autumn | female | Yes |
| Normal | Ref |  |  |  |
| Low | 0.00 (0.00-Inf) | Winter | female | Yes |
| Overweight | 1.57 (0.88-2.78) | Winter | female | Yes |
| Obesity | 3.72 (1.68-8.25) | Winter | female | Yes |
| Normal | Ref |  |  |  |
| Low | 0.00 (0.00-Inf) | Spring | male | Yes |
| Overweight | 2.04 (1.23-3.38) | Spring | male | Yes |
| Obesity | 1.74 (0.75-4.05) | Spring | male | Yes |
| Normal | Ref |  |  |  |
| Low | 0.29 (0.04-2.19) | Summer | male | Yes |
| Overweight | 1.75 (1.08-2.86) | Summer | male | Yes |
| Obesity | 2.32 (1.03-5.21) | Summer | male | Yes |
| Normal | Ref |  |  |  |
| Low | 0.73 (0.22-2.49) | Autumn | male | Yes |
| Overweight | 1.57 (0.99-2.49) | Autumn | male | Yes |
| Obesity | 4.19 (2.08-8.47) | Autumn | male | Yes |
| Normal | Ref |  |  |  |
| Low | 0.32 (0.08-1.34) | Winter | male | Yes |
| Overweight | 1.75 (1.13-2.72) | Winter | male | Yes |
| Obesity | 2.23 (1.03-4.85) | Winter | male | Yes |

*Abbreviations: GDM, gestational diabetes mellitus; CI, confidence intervals; aOR, adjusted odds ratios; Ref, reference.*
