# Supplementary material for: Protective Effect of Maternal First-Trimester Low Body Mass Index Against Macrosomia: A 10-Year Cross-Sectional Study
Source: Front Endocrinol (Lausanne). 2022 Feb 10;13:805636. doi: 10.3389/fendo.2022.805636 (PMC8866317; doi:10.3389/fendo.2022.805636)
Supplement: Supplementary file 6 [file Table_6.doc]

**TABLE S6 | STROBE Statement—Checklist of items that should be included in reports of cohort studies**

|  | Item No | Recommendation | Completed | Section | Page and Line numbers |
| --- | --- | --- | --- | --- | --- |
| **Title and abstract** | 1 | (*a*) Indicate the study’s design with a commonly used term in the title or the abstract | √ | Title; abstract | Page 1: Line 2;  Page 2: Line 46 |
|  |  | (*b*) Provide in the abstract an informative and balanced summary of what was done and what was found | √ | abstract | Page 2: Line 46-72 |
| Introduction | | | |  |  |
| Background/rationale | 2 | Explain the scientific background and rationale for the investigation being reported | √ | Introduction | Page 3: Line 88-108 |
| Objectives | 3 | State specific objectives, including any prespecified hypotheses | √ | Introduction | Page 3: Line 108-112 |
| Methods | | | |  |  |
| Study design | 4 | Present key elements of study design early in the paper | √ | Study design and data sources | Page 3: Line 115-122 |
| Setting | 5 | Describe the setting, locations, and relevant dates, including periods of recruitment, exposure, follow-up, and data collection | √ | Study design and data sources | Page 3 Line 115-126 |
| Participants | 6 | (*a*) Give the eligibility criteria, and the sources and methods of selection of participants. Describe methods of follow-up | √ | Study design and data sources | Page 3: Line 122-128 |
|  |  | (*b*) For matched studies, give matching criteria and number of exposed and unexposed |  |  |  |
| Variables | 7 | Clearly define all outcomes, exposures, predictors, potential confounders, and effect modifiers. Give diagnostic criteria, if applicable | √ | Data collection and measurements | Page 4: Line 134-141 |
| Data sources/ measurement | 8* | For each variable of interest, give sources of data and details of methods of assessment (measurement). Describe comparability of assessment methods if there is more than one group | √ | Data collection and measurements | Page 4: Line 142-158 |
| Bias | 9 | Describe any efforts to address potential sources of bias | √ | Data collection and measurements | Page 4: Line 143-147 |
| Study size | 10 | Explain how the study size was arrived at | √ | Study design and data sources | Page 3: Line 122-128 |
| Quantitative variables | 11 | Explain how quantitative variables were handled in the analyses. If applicable, describe which groupings were chosen and why | √ | Statistical analysis | Page 4: Line 160-161 |
| Statistical methods | 12 | (*a*) Describe all statistical methods, including those used to control for confounding | √ | Statistical analysis | Page 4: Line 161-169 |
|  |  | (*b*) Describe any methods used to examine subgroups and interactions | √ | Statistical analysis | Page 4: Line 169-173 |
|  |  | (*c*) Explain how missing data were addressed |  |  |  |
|  |  | (*d*) If applicable, explain how loss to follow-up was addressed |  |  |  |
|  |  | (*e*) Describe any sensitivity analyses | √ | Statistical analysis | Page 4: Line 169-173 |
| Results | | | |  |  |
| Participants | 13* | (a) Report numbers of individuals at each stage of study—eg numbers potentially eligible, examined for eligibility, confirmed eligible, included in the study, completing follow-up, and analysed | √ | Results | Page 5: Line 178-184 |
|  |  | (b) Give reasons for non-participation at each stage | √ | Results | Page 5: Line 178-179 |
|  |  | (c) Consider use of a flow diagram | √ | Figure 1 | FIGURE 1 |
| Descriptive data | 14* | (a) Give characteristics of study participants (eg demographic, clinical, social) and information on exposures and potential confounders | √ | Characteristics of participants in Results; Table 1 and Table S1 | Page 5: Line 178-184,  Table 1 and Table S1 |
|  |  | (b) Indicate number of participants with missing data for each variable of interest |  |  |  |
|  |  | (c) Summarise follow-up time (eg, average and total amount) |  |  |  |
| Outcome data | 15* | Report numbers of outcome events or summary measures over time |  |  |  |
| Main results | 16 | (*a*) Give unadjusted estimates and, if applicable, confounder-adjusted estimates and their precision (eg, 95% confidence interval). Make clear which confounders were adjusted for and why they were included | √ | Table 2 in Results | Page 5: Line 191-201, Table 2 |
|  |  | (*b*) Report category boundaries when continuous variables were categorized | √ | Results | Page 5: Line 191-201 |
|  |  | (*c*) If relevant, consider translating estimates of relative risk into absolute risk for a meaningful time period |  |  |  |
| Other analyses | 17 | Report other analyses done—eg analyses of subgroups and interactions, and sensitivity analyses | √ | Results | Page 5-6 Line 203-233 |
| Discussion | | | |  |  |
| Key results | 18 | Summarise key results with reference to study objectives | √ | Discussion | Page 6-7: Line 236-244 |
| Limitations | 19 | Discuss limitations of the study, taking into account sources of potential bias or imprecision. Discuss both direction and magnitude of any potential bias | √ | Strengths and Limitations | Page 7-8: Line 294-307 |
| Interpretation | 20 | Give a cautious overall interpretation of results considering objectives, limitations, multiplicity of analyses, results from similar studies, and other relevant evidence | √ | Strengths and Limitations | Page 7-8: Line 294-307 |
| Generalisability | 21 | Discuss the generalisability (external validity) of the study results | √ | Strengths and Limitations and Conclusion | Page 78: Line 286-317 |
| Other information | | | |  |  |
| Funding | 22 | Give the source of funding and the role of the funders for the present study and, if applicable, for the original study on which the present article is based | √ | Acknowledgements | Page 8: Line 332-335 |

*Give information separately for exposed and unexposed groups.
